# C-Reactive Protein Binds to Cholesterol Crystals and Co-Localizes with the Terminal Complement Complex in Human Atherosclerotic Plaques

**DOI:** 10.3389/fimmu.2017.01040

**Published:** 2017-08-29

**Authors:** Katrine Pilely, Stefano Fumagalli, Anne Rosbjerg, Ninette Genster, Mikkel-Ole Skjoedt, Carlo Perego, Angela M. R. Ferrante, Maria-Grazia De Simoni, Peter Garred

**Affiliations:** ^1^Laboratory of Molecular Medicine, Department of Clinical Immunology, Section 7631, Faculty of Health and Medical Sciences, Rigshospitalet, University of Copenhagen, Copenhagen, Denmark; ^2^Istituto di Ricerche Farmacologiche Mario Negri, Department of Neuroscience, IRCCS, Milan, Italy; ^3^Università Cattolica del S.Cuore, Istituto di Patologia Chirurgica, Fondazione “Policlinico Universitario A. Gemelli”, Polo Scienze cardiovascolari e toraciche, Roma, Italy

**Keywords:** atherosclerosis, complement activation, cholesterol crystals, C-reactive protein, pentraxin 3, serum amyloid P component, c1q, membrane attack complex, C3

## Abstract

Inflammation is a part of the initial process leading to atherosclerosis and cholesterol crystals (CC), found in atherosclerotic plaques, which are known to induce complement activation. The pentraxins C-reactive protein (CRP), long pentraxin 3 (PTX3), and serum amyloid P component (SAP) are serum proteins associated with increased risk of cardiovascular events and these proteins have been shown to interact with the complement system. Whether the pentraxins binds to CC and mediate downstream complement-dependent inflammatory processes remains unknown. Binding of CRP, PTX3, and SAP to CC was investigated *in vitro* by flow cytometry and fluorescence microscopy. CRP, PTX3, and SAP bound to CC in a concentration-dependent manner. CRP and PTX3 interacted with the complement pattern recognition molecule C1q on CC by increasing the binding of both purified C1q and C1q in plasma. However, CRP was the strongest mediator of C1q binding and also the pentraxin that most potently elevated C1q-mediated complement activation. In a phagocytic assay using whole blood, we confirmed that phagocytosis of CC is complement dependent and initiated by C1q-mediated activation. The pathophysiological relevance of the *in vitro* observations was examined *in vivo* in human atherosclerotic plaques. CRP, PTX3, and SAP were all found in atherosclerotic plaques and were located mainly in the cholesterol-rich necrotic core, but co-localization with the terminal C5b-9 complement complex was only found for CRP. In conclusion, this study identifies CRP as a strong C1q recruiter and complement facilitator on CC, which may be highly relevant for the development of atherosclerosis.

## Introduction

Vascular diseases caused by atherosclerosis are the leading cause of death and morbidity worldwide, with ischemic heart disease and stroke accounting for 15 million deaths in 2015 (1). Atherosclerosis is a chronic inflammatory disease, where lipids and cholesterol builds up in the arterial wall, causing the formation of a plaque. A hallmark of atherosclerotic plaques, also found in early atherosclerotic lesions, is deposition of solid phase cholesterol crystals (CCs) (2). CCs are known to induce inflammation *via* activation of the complement system (3–6) leading to a subsequent cytokine release and activation of the nod-like receptor pyrin domain-containing 3 (NLRP3) inflammasome (2, 7–9). The complement system is an important part of the innate immune system. The complement system is initiated through three pathways; the classical pathway, the lectin pathway, and the alternative pathway and is initiated when pathway-specific pattern recognition molecules (PRMs) recognize pathogen-associated molecular patterns or damage-associated molecular patterns. CCs are known to activate all three pathways (6, 8). Activation initiates a cascade reaction leading to the cleavage of C3 into C3a and C3b and eventually the cleavage of C5 into C5a and C5b and the formation of a terminal C5b-9 complement complex (TCC). Activation results in opsonization and phagocytosis of pathogens or damaged self-structures through deposition of C3b, and pro-inflammatory signaling induced by C3a and C5a (10).

The pentraxins are a family of proteins consisting of the long pentraxin 3 (PTX3) and the short pentraxins C-reactive protein (CRP) and serum amyloid P component (SAP) (11). CRP and PTX3 are acute-phase proteins in humans and are expressed in response to inflammatory stimulation, whereas SAP is constitutively present in human blood in a concentration of 30–50 mg/L (12). CRP is predominantly expressed in the liver and in response to systemic inflammatory mediators, mainly IL-6, CRP median blood concentration increases from 0.8 mg/L to more than 500 mg/L (13). PTX3 is expressed in the tissue by macrophages, dendritic cells, and endothelial cells and is released from neutrophil intracellular granules at sites of infection upon primary inflammatory stimuli, e.g., TLR engagement, TNFα, and IL-1β (14–18). In healthy individuals, plasma PTX3 levels are below 2 µg/L, but increase rapidly upon inflammatory stimuli to more than 100 µg/L (11). The pentraxins interact with several complement PRMs as well as complement regulators. PTX3, CRP, and SAP are able to recruit the classical pathway PRM C1q, thereby inducing complement activation and phagocytosis (19–22), and have also been shown to interact with lectin pathway PRMs mannose-binding lectin (MBL) and the Ficolins (23–26). Furthermore, PTX3 and CRP bind the complement regulators factor H and C4b-binding protein (C4BP) (27–30) and SAP binds C4BP (31). Both CRP and PTX3 are not only biomarkers of inflammation but have also been associated with increased risk of cardiovascular disease. High-sensitivity CRP (hsCRP) is an established risk biomarker of myocardial ischemia and infarction, unstable angina, and chronic atherosclerotic disease and is used as a risk biomarker of first and recurrent cardiovascular events (32–40). PTX3 is associated with severity and mortality of acute myocardial infarction and cardiovascular outcomes, and risk of cardiac events in patients with heart failure (41–48). Also, increased levels of SAP have been associated with cardiovascular disease (49). Previously PTX3, CRP and SAP have been found in human atherosclerotic plaques (50–53) and CC, similar to those found in atherosclerosis, and are known to activate the complement system and induce inflammation. The pentraxins are known to interact with the complement system, especially C1q, but whether they interact with CC is unknown.

Thus, the purpose of this study was to investigate the binding of PTX3, CRP, and SAP to CC *in vitro* and to study their interaction with C1q on the surface of the CC. Furthermore, we wanted to investigate the pentraxins in CC-induced complement activation both *in vitro* and by studying their co-localization with complement deposition in atherosclerotic plaques. This would support our hypothesis that binding of the pentraxins to CC could be a key link between complement activation, inflammation, and atherosclerosis.

## Materials and Methods

### Materials

#### Reagents

Ultrapure cholesterol (C8667), 1-propanol (279544), human serum albumin (HSA) (A9731), RPMI 1640 medium (R5886), and bovine serum albumin (BSA) (A2153) were purchased from Sigma-Aldrich. EDTA (324503), SAP purified from human serum (565190), and CRP purified from human ascites (236600) were all from Calbiochem. Purified human C1q (A099) were purchased from Complement Technology, Lysing buffer (S2364) was from DAKO, ProLong^®^ Diamond Antifade Mountant (P36965) was from Molecular Probes by Life Technologies, TSA amplification kit was from Perkin Elmer, and True-Black quencher was from Biotium.

#### Commercial Antibodies

Rabbit anti-human SAP polyclonal antibody (pAb) (565191, Calbiochem), rabbit anti-human CRP pAb (235752, Calbiochem), rabbit anti-human C1q pAb (A0136, Dako), mouse anti-human complement component C5b-9 monoclonal antibody (mAb) (IgG2a) (011-01, Antibody Shop), mouse IgG1 isotype control (BD Biosciences), mouse IgG2a isotype control (BD Biosciences), rabbit IgG isotype control (Invitrogen), FITC-conjugated goat anti-rabbit pAb (F1262, Sigma-Aldrich), FITC-conjugated goat anti-mouse pAb (F0479, Dako), mouse anti-human CD45 FITC/CD14 PE (342408, BD Biosciences), mouse anti-human CD11b APC-Cy7 (560914, BD Biosciences), Alexa-555-conjugated goat anti-mouse IgG (A-21424, Thermo Fischer), biotinylated goat anti-rabbit IgG (BA-1000, Vector Laboratories), or biotinylated goat anti-mouse IgG (BA-9200, Vector Laboratories) are the commercial antibodies.

#### In-House-Produced Antibodies

Mouse anti-PTX3 mAbs clone 11-19-20, 11-19-66, 11-19-61, and 11-19-73 are in-house Abs produced as previously described (54).

#### Complement Inhibitors

C3 inhibitory peptide; Compstatin Cp40 (55), C5 inhibitor; Eculizumab (Soliris, Alexion Pharmaceuticals), C1q inhibitory mouse anti-human C1q mAb clone 85 (IgG1) (MW1828, Sanquin) are the complement inhibitors.

### CC Precipitation

Cholesterol crystals were generated essentially as described by Samstad et al. (8). 2 mg/mL ultrapure cholesterol was dissolved in 1-propanol. CCs were precipitated by adding distilled water (1:1.5). The solution was left undisturbed for 15 min for the crystals to stabilize. CCs were collected by centrifugation and the remaining 1-propanol was removed by evaporation. All steps were performed at room temperature (RT). The CC were resuspended in phosphate-buffered saline (PBS) with 0.05% HSA and stored at 4°C in the dark.

### Production of Recombinant PTX3 (rPTX3)

Recombinant PTX3 was produced as previously described (54, 56). In short, PTX3 was expressed in CHO-DG44 cells cultivated in RPMI 1640 medium supplemented with 10% heat-inactivated fetal calf serum (FCS), 100 U/mL penicillin, 0.1 mg/mL streptomycin, 2 mM l-glutamine, and 200 nM methotrexate. Culture supernatant was harvested from stable transfected CHO cells. rPTX3 was purified from culture supernatant by affinity chromatography using a mixture of in-house-produced anti-PTX3 mAbs (clone 11-19-20, 11-19-61, 11-19-66, 11-19-73). Western blot of purified rPTX3 is found as Figure S1 in Supplementary Material.

### Collection of Plasma and Serum Samples

A pool of normal human plasma (NHP) or normal human serum (NHS) was obtained by drawing venous blood from six healthy donors (three male and three female donors) into hirudin vials or vials with no additive, respectively. Plasma was collected immediately from the hirudin blood samples by centrifugation at 3000 × *g* for 15 min. Blood samples for serum collection were left at RT for 2 h, before the serum was collected by centrifugation at 3000 × *g* for 15 min. Serum samples from three individuals with systemic inflammation were collected and stored as individual serum samples until further analysis. A pool of umbilical cord serum (UCS) was obtained from umbilical cord blood collected from three individuals. Plasma and serum samples were stored at −80°C until further analysis.

### Flow Cytometry

Samples were analyzed by flow cytometry using a Gallios flow cytometer (Beckman Coulter) and analyzed using Kaluza software (Beckman Coulter).

#### Binding of PTX3, CRP, SAP, and C1q to CC

Cholesterol crystals were incubated with rPTX3, purified CRP (pCRP), purified SAP (pSAP), purified C1q (pC1q), and plasma or serum samples diluted in Barbital buffer (5 mM Barbital sodium, 145 mM NaCl, 2 mM CaCl_2_, 1 mM MgCl_2_, pH 7.4) with 0.5% BSA (Barbital/BSA) ± 10 mM EDTA for 30 min at 37°C. Antigen binding to CC was detected by incubating CC with antigen-specific antibodies or isotype controls: mouse anti-PTX3 mAb 11-19-66 (5 µg/mL), rabbit anti-CRP pAb (0.1 µg/mL), rabbit anti-SAP pAb (0.05 µg/mL), rabbit anti-C1q pAb (0.5 µg/mL), mouse IgG1 (5 µg/mL), or rabbit IgG (0.05, 0.1, or 0.5 µg/mL) for 30 min at 4°C. CCs were then incubated with secondary antibodies; FITC-conjugated goat anti-mouse IgG or FITC-conjugated goat anti-rabbit IgG. CCs were washed in Barbital buffer with 0.5% FCS (Barbital/FCS) by centrifugation (5 min, 500 × *g*) between each step.

#### Enhanced Binding of PTX3, CRP, or SAP by Preincubation of CC with C1q

Cholesterol crystals were preincubated with pC1q diluted in Barbital/BSA for 30 min at 37°C. CCs were then washed in Barbital/BSA and incubated with rPTX3, pCRP, or pSAP for 30 min at 37°C. PTX3, CRP, and SAP binding to CC were detected as described above.

#### Enhanced Binding of C1q by Preincubation of CC with PTX3, CRP, or SAP

Cholesterol crystals were preincubated with rPTX3, pCRP, or pSAP diluted in Barbital/BSA for 30 min at 37°C. CCs were then washed in Barbital/BSA and incubated with pC1q, 5% NHP, or 5% UCS for 30 min at 37°C. C1q binding to CC was detected as described above.

#### Increased Complement Activation on CC by Preincubation with PTX3, CRP, or SAP

Cholesterol crystals were preincubated with rPTX3, pCRP, or pSAP diluted in Barbital/BSA for 30 min at 37°C. CCs were then washed in Barbital/BSA and incubated with 5 or 10% NHP or UCS for 30 min at 37°C. Complement activation was inhibited by preincubating NHP or UCS with C1q inhibitory antibody and mouse anti-human C1q clone 85 (IgG1) or a mouse IgG1 isotype control. C5b-9 deposition on CC was detected using mouse anti-human complement component C5b-9 mAb followed by FITC-conjugated goat anti-mouse IgG.

#### Phagocytosis of CC

Hirudin whole blood was collected from three healthy donors and preincubated for 5 min at RT with 20 mM C3 inhibitor; Compstatin, Cp40, 50 µg/mL C5 inhibitor; Eculizumab, 50 µg/mL C1q inhibitory antibody; and mouse anti-human C1q clone 85 (IgG1) or a mouse IgG1 isotype control. Whole blood was then stimulated with PBS or CC for 30 min at 37°C. Cells were stained with anti-CD45-FITC/CD14-PE and anti-CD11b APC-Cy7 for 20 min at RT in the dark. Red blood cells were lysed with DAKO lysing buffer for 15 min at RT and cells were washed three times in PBS with 0.5% FCS before analysis. Phagocytosis was determined as percentage of cells phagocytosing CC, determined as a shift in side scatter, and by the expression of complement receptor 3 (CR3/CD11b) measured as median fluorescence intensity. Granulocytes and monocytes were gated based on CD14 expression.

### Microscopy of CCs

Cholesterol crystals were incubated with rPTX3, pCRP, pSAP, and plasma or serum samples diluted in Barbital/BSA for 30 min at 37°C. Antigen binding to CC was detected by incubating CC with antigen-specific antibodies or isotype controls: mouse anti-PTX3 mAb 11-19-66 (5 µg/mL), rabbit anti-CRP pAb (0.1 µg/mL), rabbit anti-SAP pAb (0.05 µg/mL), mouse IgG1 (5 µg/mL), and rabbit IgG (0.05 or 0.1 µg/mL) for 30 min at 4°C. CCs were then incubated with secondary antibodies: FITC-conjugated goat anti-mouse IgG or FITC-conjugated goat anti-rabbit IgG. CCs were then washed in Barbital/FCS with centrifugation (5 min, 500 × *g*) between each step. CCs were placed on slides with centrifugation for 5 min at 500 × *g* (cytospin) and mounted with ProLong^®^ Diamond Antifade Mountant. Microscopy was performed using a Zeiss Axio Observer through a 63×/1.40 oil DIC Plan-Apochromat objective. Imaging conditions were kept constant when acquiring images to be compared.

### Immunofluorescence of Human Atherosclerotic Plaques

#### Patients and Sampling Processing

Plaques were obtained from patients undergoing carotid endarterectomy referring to department of Vascular Surgery at A. Gemelli Hospital during 2013–2015 (57). Immediately after surgery specimens were snap-frozen. The plaques were cut coronally in 20-µm sections with a cryostate. Cutting was done at the site of the maximal plaque thickening ±1 mm (58). Sections were laid on a gelatinized glass and before immunofluorescence, thawed by 5 min washing with 0.05 M tris-buffered saline at RT, and then post-fixated by 15 min incubation with 4% paraformaldehyde.

#### Immunofluorescence and Confocal Microscopy Analysis

After thorough washings with 0.01 M PBS, sections were incubated with blocking solution (10% normal goat serum, 0.3% Triton X-100) for 1 h at RT and then with primary antibodies in the same solution overnight at 4°C. Primary monoclonal antibodies used were: mouse anti-human C5b-9 (10 µg/mL), mouse anti-human PTX3 11-19-66 (9 µg/mL), rabbit anti-human CRP (70 µg/mL), and rabbit anti-human SAP (10 µg/mL). Sections were then incubated with Alexa-555 conjugated secondary antibody goat anti-mouse IgG (4 µg/mL) for 1.5 h at RT or biotinylated secondary antibody goat anti-rabbit IgG or Goat anti-mouse IgG (7.5 µg/mL) for 1 h at RT, followed by fluorescent signal coupling with streptavidin TSA amplification kit (fluorescein). Sections were then incubated with True-Black quencher (1:20 in 70% Ethanol) to quench non-specific fluorescent signals. Appropriate negative controls without the primary antibodies were performed. None of the immunofluorescence reactions revealed unspecific fluorescent signal in the negative controls (Figure S4 in Supplementary Material). To avoid a cross signal in the double immunofluorescence for PTX3 and C5b-9 (both antibodies were developed in mouse), we tested different working dilutions of the anti-human PTX3 antibody and chose the lowest concentration providing: (1) a visible signal with fluorescein at excitation wavelength 488 nm; (2) no signal with Alexa-555 at excitation wavelength 532 nm. Thus, Alexa-555 fluorescent signal is selectively associated with C5b-9 expression (59).

Immunofluorescence was acquired using a scanning sequential mode to avoid bleed-through effects by an IX81 microscope equipped with a confocal scan unit FV500 with three laser lines: Ar–Kr (488 nm), He–Ne red (646 nm), and He–Ne green (532 nm, Olympus) and a UV diode. High magnification three-dimensional images were acquired over a 10 µm *z*-axis with a 0.46-µm step size and processed using Imaris software (Bitplane). Images of the whole plaques were acquired at 10× (pixel size of 0.646 µm) with an Olympus BX-61 Virtual Stage microscope to have completed stitching of the whole plaque. Exclusion images were obtained by Fiji software image calculator protocol (“subtract” function). Images were finally managed using GIMP software.

### Ethical Approvals

The study was approved by the regional Health Ethics Committee in the Capital Region of Denmark (reference no. H2-2011-133). Retrospective examination of carotid plaques was approved by the local ethical committee Board; Comitato Etico della Fondazione Policlinico Universitario A. Gemelli (reference no. 26089/16).

### Statistical Analysis

GraphPad Prism version 6 (Graphpad Software) was used for statistical analysis. Statistical analysis was performed on three independent experiments or on three individual healthy donors (phagocytosis of CC) using two-tailed paired *t*-test make a direct comparison between each experimental setup. A *p* value <0.05 was considered statistically significant.

## Results

### Binding of Recombinant, Purified, and Native PTX3, CRP, and SAP to CCs

**Figure 1:**
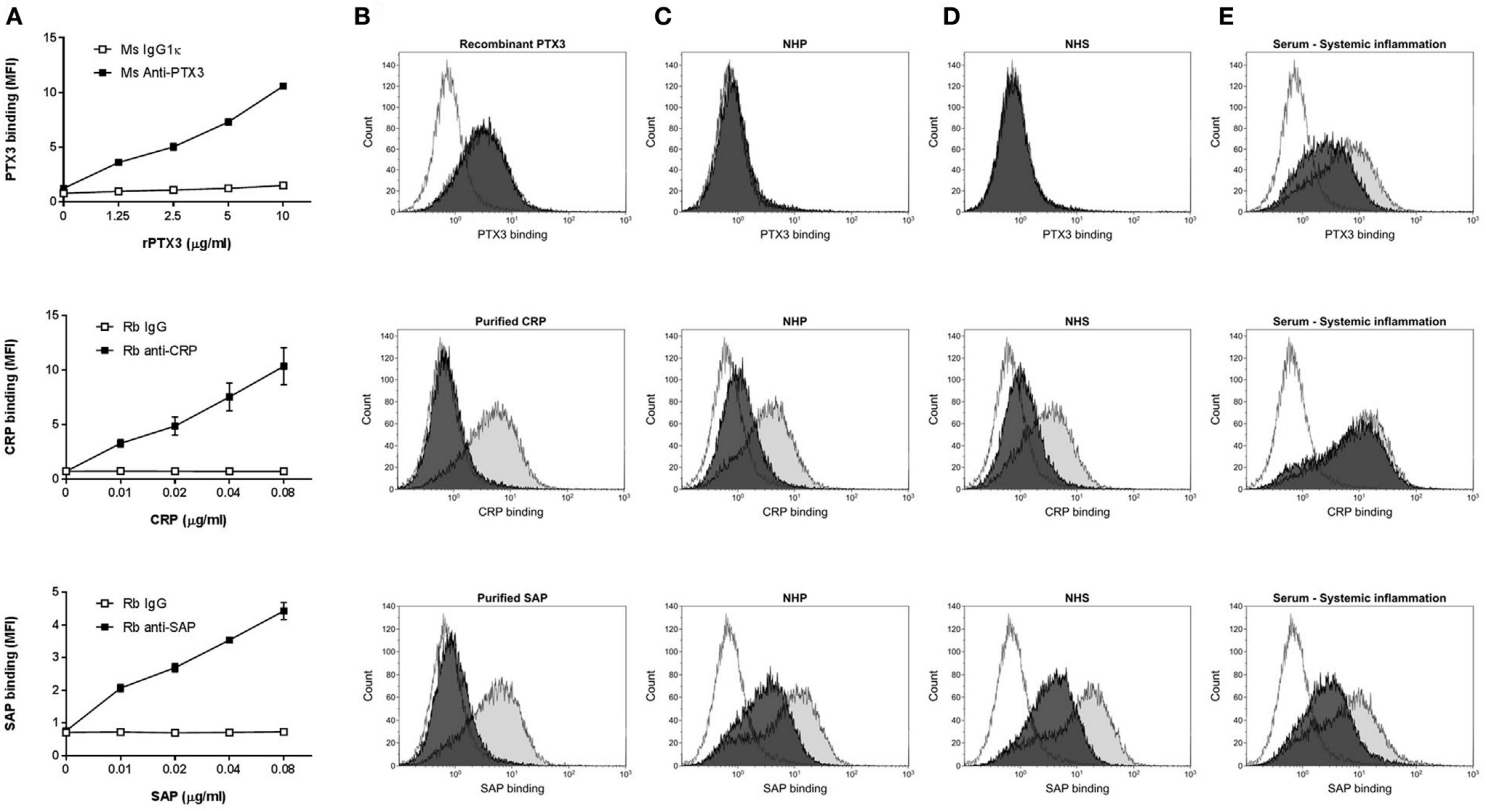
Binding of recombinant, purified, and native pentraxin 3 (PTX3), C-reactive protein (CRP), or SAP to CCs. **(A)** Concentration-dependent binding of recombinant PTX3 (rPTX3), purified CRP (pCRP), and purified SAP (pSAP) to CC. Binding was assessed by flow cytometry as MFI and data are given as mean ± SEM (*n* = 3). **(B)** Binding of rPTX3, pCRP, or pSAP to CC ± 10 mM EDTA (black/gray). **(C–E)** Binding of native PTX3, CRP, or SAP from 5% NHP **(C)**, NHS **(D)** or a pool of serum from three individuals with systemic inflammation **(E)** diluted in Barbital/BSA ± 10 mM EDTA (black/gray). Isotype controls are shown as white histograms. Histograms represent one of three independent experiments. CCs, cholesterol crystals; NHP, normal human plasma; NHS, normal human serum; MFI, median fluorescence intensity.

**Figure 2:**
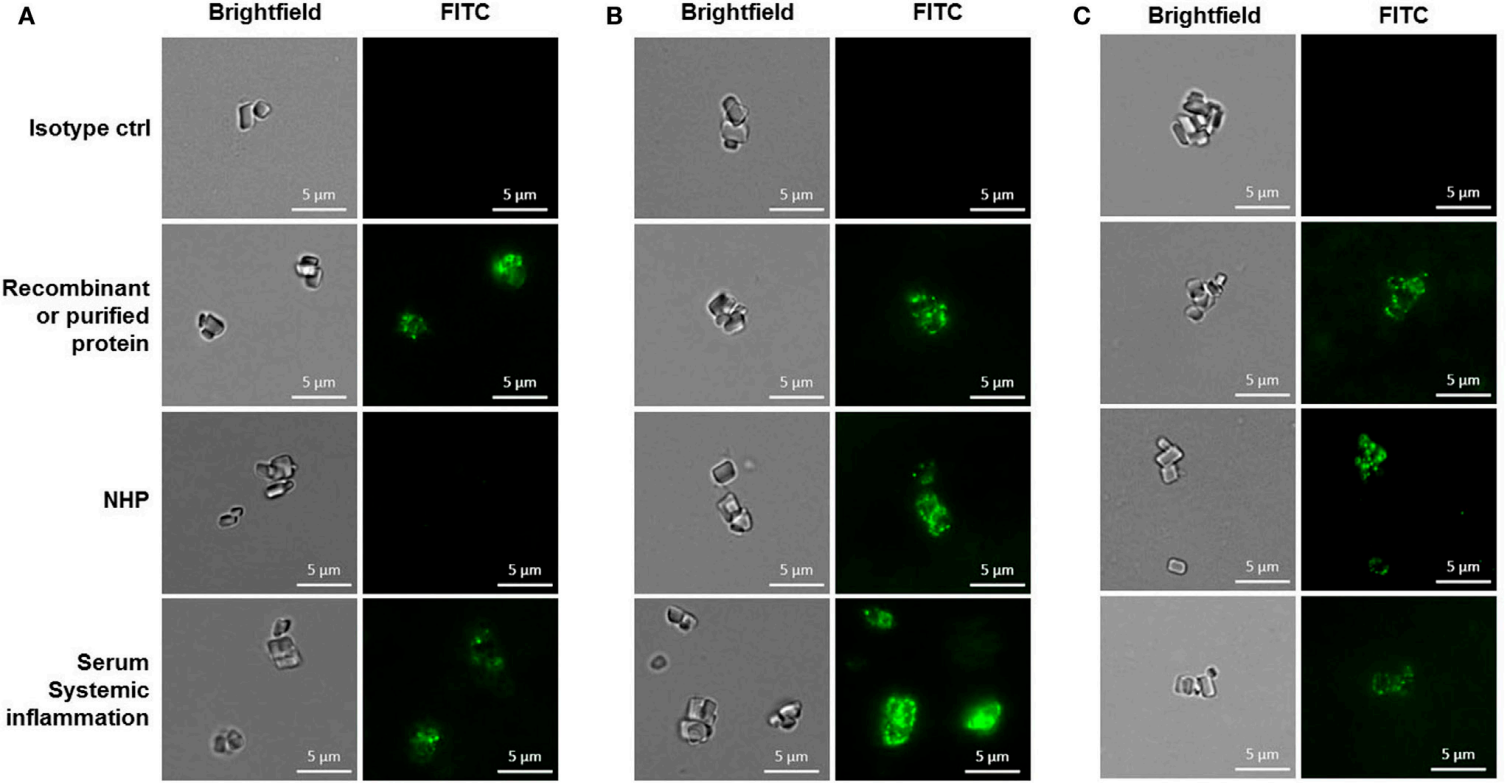
Binding of pentraxin 3 **(A)**, C-reactive protein **(B)**, or SAP **(C)** to cholesterol crystals assessed by fluorescence microscopy. Imaging conditions were kept constant when acquiring images to be compared. Results are representative of three independent experiments. NHP, normal human plasma.

We investigated the binding of the pentraxins: PTX3, CRP, or SAP to CC *in vitro* by flow cytometry (Figure 1) and fluorescence microscopy (Figure 2). rPTX3, pCRP, and pSAP bound to CC in a concentration-dependent manner (Figure 1A); however 100-fold higher concentrations of rPTX3 were necessary to obtain binding than for pCRP and pSAP. Binding of rPTX3 was not inhibited by EDTA, whereas binding of pCRP and SAP was highly calcium dependent and was completely inhibited by addition of EDTA (Figure 1B). Binding of native PTX3, CRP, and SAP was investigated by incubating CC with 5% NHP, NHS, or a pool of serum from three individuals with systemic inflammation (Figures 1C–E). As expected, no binding of PTX3 to CC measured by flow cytometry was detected from NHP or NHS with PTX3 concentrations below detection limit (<2 µg/L) in ELISA (Figures 1C,D). Binding of native PTX3 to CC was observed from a pool of serum from individuals with systemic inflammation (mean PTX3 concentration of 33.0 µg/L) (Figure 1E). It is noteworthy that the binding of native serum PTX3 required a substantially smaller amount of protein compared to the binding of rPTX3 where no serum factors were present (Figure 1A). Native CRP bound to CC from NHP, NHS, and a pool of serum from three individuals with systemic inflammation (Figures 1C–E). Binding of CRP was increased in serum from individuals with systemic inflammation (mean CRP concentration of 99.5 mg/L) compared to NHP (CRP concentration of 0.88 mg/L) or NHS (CRP concentration of 0.93 mg/L). Native SAP bound to CC from NHP, NHS, and serum from individuals with systemic inflammation (Figures 1C–E). Binding of native PTX3, CRP, and SAP to CC was not completely inhibited by EDTA (Figures 1C–E). Altogether, data show that PTX3, CRP, and SAP bind specifically to CC as recombinant proteins, purified native proteins and non-purified proteins in plasma or serum samples. Plasma and serum concentrations of PTX3 and CRP in the different samples measured by ELISA or established CRP and hsCRP assays are found in Table S1 in Supplementary Material. Binding of PTX3, CRP, and SAP with/without calcium to CC from the three individuals with systemic inflammation is found as Figure S2 in Supplementary Material.

### PTX3, CRP, and C1q Interact on the Surface of CCs

**Figure 3:**
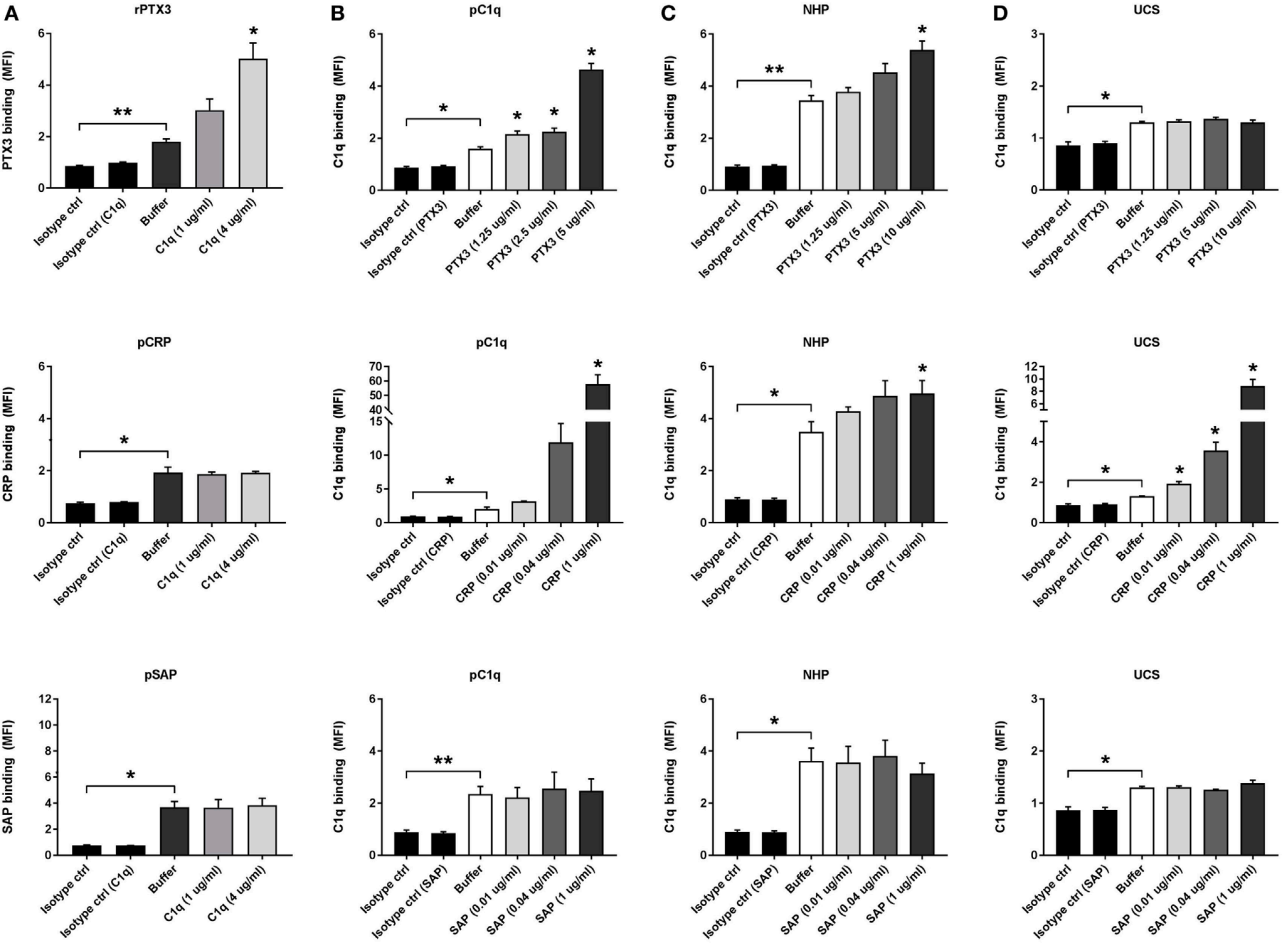
Pentraxin 3 (PTX3), C-reactive protein (CRP), and C1q interact on the surface of CCs. C1q binding to CC increase binding of PTX3 **(A)** and binding of PTX3 or CRP increase the binding of purified C1q (pC1q) and native C1q from plasma or serum samples **(B–D)**. **(A)** Binding of recombinant PTX3 (rPTX3) (2.5 µg/mL), purified CRP (pCRP) (0.02 µg/mL), or purified SAP (pSAP) (0.02 µg/mL) to CC preincubated with 0, 1, or 4 µg/mL pC1q. **(B)** Binding of pC1q (2 µg/mL) to CC preincubated with rPTX3 (0, 1.25, 2.5 or 5 µg/mL), pCRP (0, 0.01, 0.04, or 1 µg/mL), or pSAP (0, 0.01, 0.04, or 1 µg/mL). **(C)** Binding of C1q from 5% NHP to CC preincubated with rPTX3 (0, 1.25, 5 or 10 µg/mL), pCRP (0, 0.01, 0.04, or 1 µg/mL), or pSAP (0, 0.01, 0.04, or 1 µg/mL). **(D)** Binding of C1q from 5% UCS to CC preincubated with rPTX3 (0, 1.25, 5, or 10 µg/mL), pCRP (0, 0.01, 0.04, or 1 µg/mL), or pSAP (0, 0.01, 0.04, or 1 µg/mL). Binding was assessed by MFI and data are given as mean ± SEM (*n* = 3). *p < 0.05, **p < 0.01 compared with buffer or as otherwise indicated. CC, cholesterol crystals; NHP, normal human plasma; UCS, umbilical cord serum; MFI, median fluorescence intensity.

To further elucidate the role of the pentraxins in CC-mediated inflammation, we investigated their interaction with the complement PRM C1q. Interactions between rPTX3, pCRP, pSAP, and pC1q were characterized by ELISA before use in flow cytometry experiments with CC to validate the functionality of the recombinant and purified proteins (Figure S3 in Supplementary Material). Preincubation of CC with pC1q significantly increased the binding of rPTX3 (2.5 µg/mL) but had no significant effect on the binding of pCRP (0.02 µg/mL) or pSAP (0.02 µg/mL) to CC (Figure 3A). Experiments where CC were preincubated with pC1q was also performed using 0.1, 0.5, and 1 µg/mL CRP or SAP, obtaining similar results (data not shown). Preincubation of CC with rPTX3 or pCRP significantly increased the binding of pC1q and native C1q from 5% NHP (Figures 3B,C). It has previously been shown that C1q binds to CC *via* IgM (6); therefore, we investigated the C1q/pentraxin interactions in UCS because here IgM is present in a concentration of 5–20% of normal adult values (60). Preincubation with pCRP significantly increased the binding of native C1q, which was not the case for PTX3 and SAP (Figure 3D). These data show that both PTX3 and CRP interact with C1q on CC, but in a different manner; PTX3 likely depends on the presence of IgM to recruit C1q, whereas CRP alone is an effective C1q recruiter. This led us to investigate the effect of PTX3 and CRP on C1q-mediated complement activation.

### Binding of CRP to CCs Increases C1q-Mediated Complement Activation in UCS

**Figure 4:**
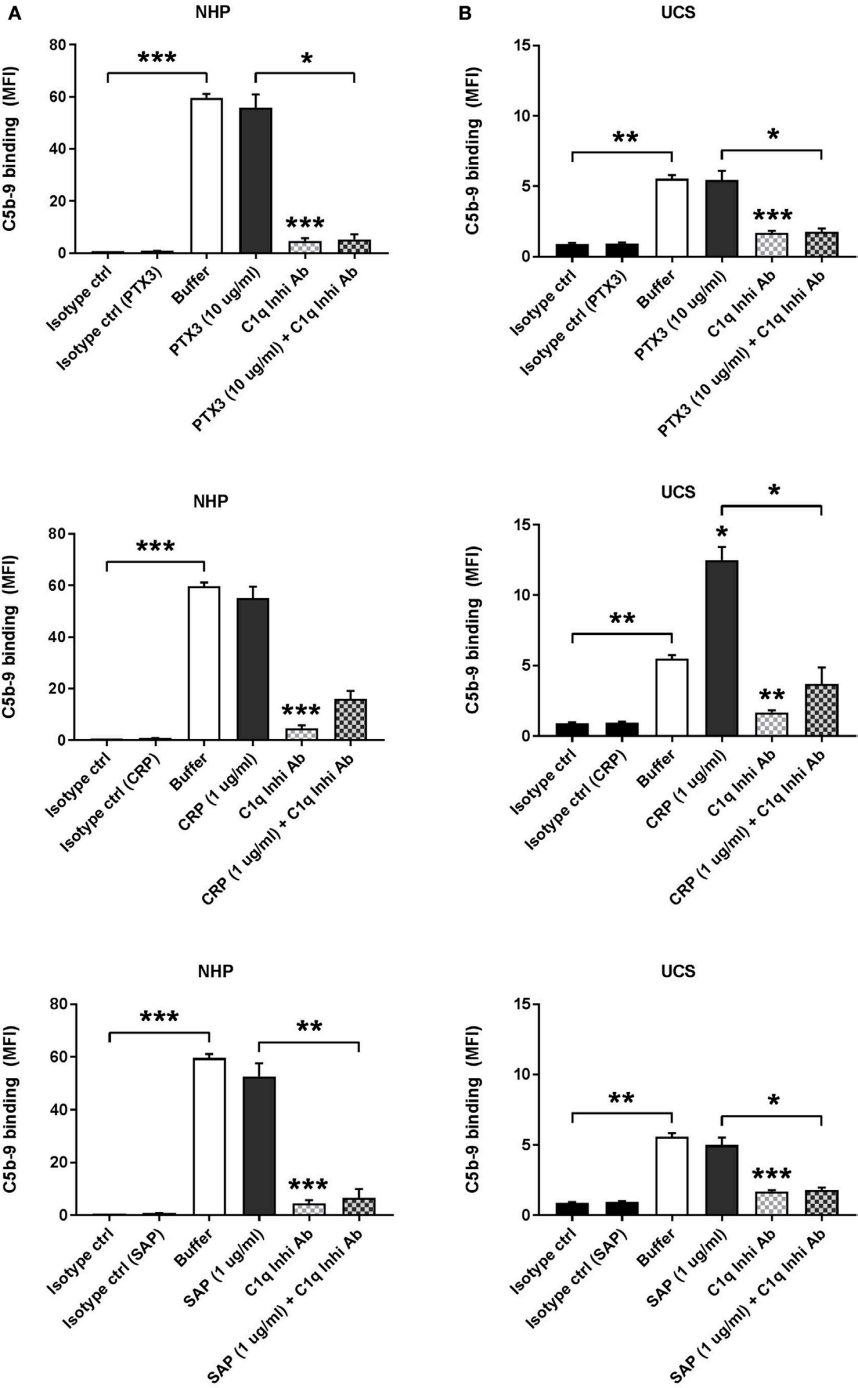
Binding of C-reactive protein (CRP) to CCs increases C1q-mediated complement activation in UCS. Complement activation was measured as C5b-9 deposition on CC and assessed by flow cytometry. **(A)** C5b-9 deposition on CC incubated with 10% NHP ± C1q inhibitory antibody (C1q Inhib Ab). CC were preincubated with recombinant PTX3 (rPTX3) (0 or 10 µg/mL), purified CRP (pCRP) (0 or 1 µg/mL), or purified SAP (pSAP) (0 or 1 µg/mL). **(B)** C5b-9 deposition on CC incubated with 10% UCS ± C1q Inhi Ab. CC were preincubated with rPTX3 (0 or 10 µg/mL), pCRP (0 or 1 µg/mL), or pSAP (0 or 1 µg/mL). Binding was assessed by MFI, and data are given as mean ± SEM (*n* = 3). *p < 0.05, **p < 0.01, ***p < 0.001 compared with buffer or as otherwise indicated. CC, cholesterol crystals; NHP, normal human plasma; UCS, umbilical cord serum; MFI, median fluorescence intensity.

CC-induced complement activation, measured as terminal C5b-9 complement complex deposition, was assessed by incubation of CC with 10% NHP (Figure 4A) or UCS (Figure 4B). CCs were preincubated with rPTX3, pCRP, or pSAP and in some experiments C1q-mediated complement activation was inhibited by preincubation of NHP or UCS with a C1q inhibitory antibody. Preincubation with rPTX3, pCRP, or pSAP did not increase C5b-9 deposition on CC incubated with NHP (Figure 4A). Preincubation of CC with pCRP significantly increased the C5b-9 deposition from UCS (Figure 4B), corresponding to the significantly increased C1q binding from UCS to CC preincubated with pCRP (Figure 3D). The increased complement activation mediated by CC preincubated with pCRP was significantly inhibited by the C1q inhibitory antibody, indicating that the increase in C5b-9 deposition on CC was due to increased C1q binding (Figure 4B). All experiments were also conducted using 5% NHP, NHS, or UCS obtaining similar results (data not shown).

### Phagocytosis of CCs by Granulocytes and Monocytes in Whole Blood Is Complement Dependent and Depends on C1q Binding

**Figure 5:**
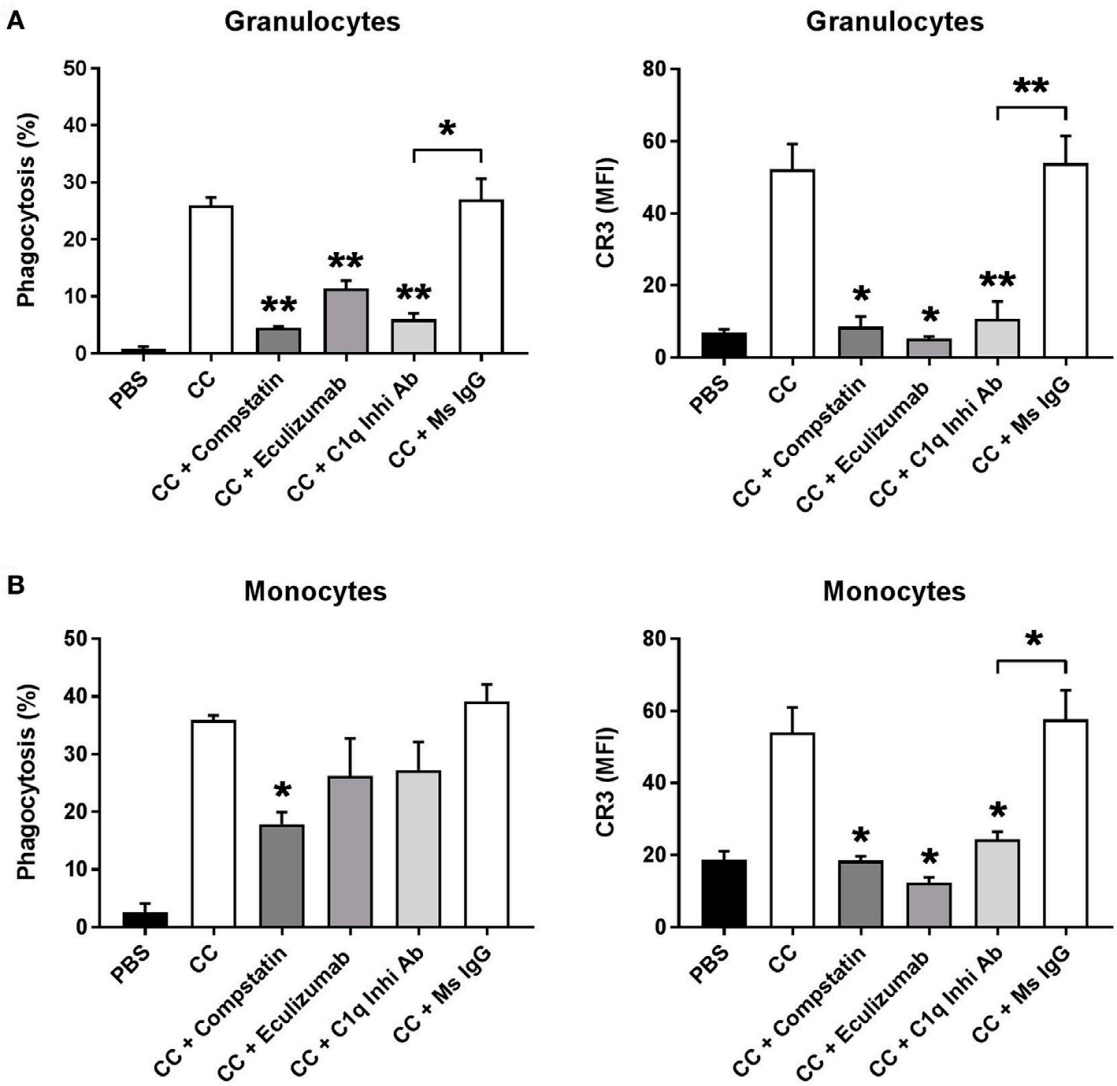
Phagocytosis of CCs by granulocytes **(A)** and monocytes **(B)** in whole blood is complement dependent and depends on C1q binding. Hirudin whole blood, preincubated 5 min with the C3 inhibitor Compstatin, Cp40, the C5 inhibitor Eculizumab, a C1q inhibitory antibody (C1q Inhib Ab), or a mouse antibody as control (Ms IgG). Whole blood was stimulated with PBS or CC. Phagocytosis was determined as percentage of cells phagocytosing CC determined as a shift in side scatter and by the expression of CR3 measured as MFI. Granulocytes and monocytes were gated based on CD14 expression. Data are given as mean ± SD (*n* = 3 healthy donors). *p < 0.05, **p < 0.01 compared with CC or as otherwise indicated. CC, cholesterol crystals; CR3, complement receptor 3; MFI, median fluorescence intensity; PBS, phosphate-buffered saline.

We investigated the role of C1q in the phagocytosis of CC. Human whole blood was preincubated with complement inhibitors Compstatin (a C3 inhibitory peptide), Eculizumab (a C5 inhibitory antibody), a C1q inhibitory antibody, or mouse IgG as control and then stimulated with PBS or CC. Compstatin, Eculizumab, and the C1q inhibitory antibody significantly reduced the percentage of granulocytes internalizing and/or binding to CC. The inhibitors also reduced the cell surface expression of the complement receptor 3 (CR3/CD11b) on both granulocytes (Figure 5A) and monocytes (Figure 5B). Compstatin, Eculizumab, and the C1q inhibitory antibody also reduced the percentage of monocytes internalizing and/or binding CC, although only the reduction using Compstatin was statistically significant. These results confirm previous reports: C1q-mediated complement activation on CC is highly important for the phagocytosis of CC by granulocytes and monocytes.

### PTX3, CRP, and SAP are Found in Human Atherosclerotic Plaques and Co-Localize with C5b-9

**Figure 6:**
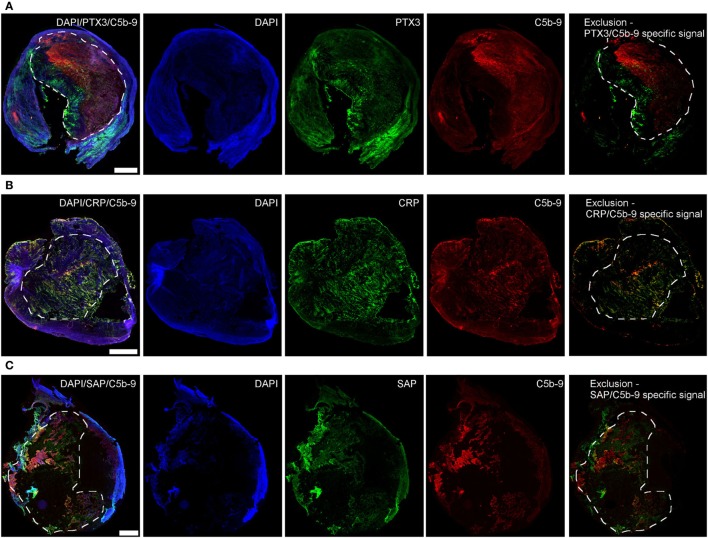
Pentraxin 3 (PTX3) **(A)**, C-reactive protein (CRP) **(B)**, and SAP **(C)** are found in human atherosclerotic plaques. PTX3, CRP, and SAP (green) deposited mainly in the necrotic core (dashed areas) where C5b-9 (red) was also mostly found. The exclusion images showed the specific signal for each of the proteins obtained after deletion of the collagen-associated unselective fluorescence. Images are representative of four plaques analyzed per marker. Scale bars = 1 mm.

**Figure 7:**
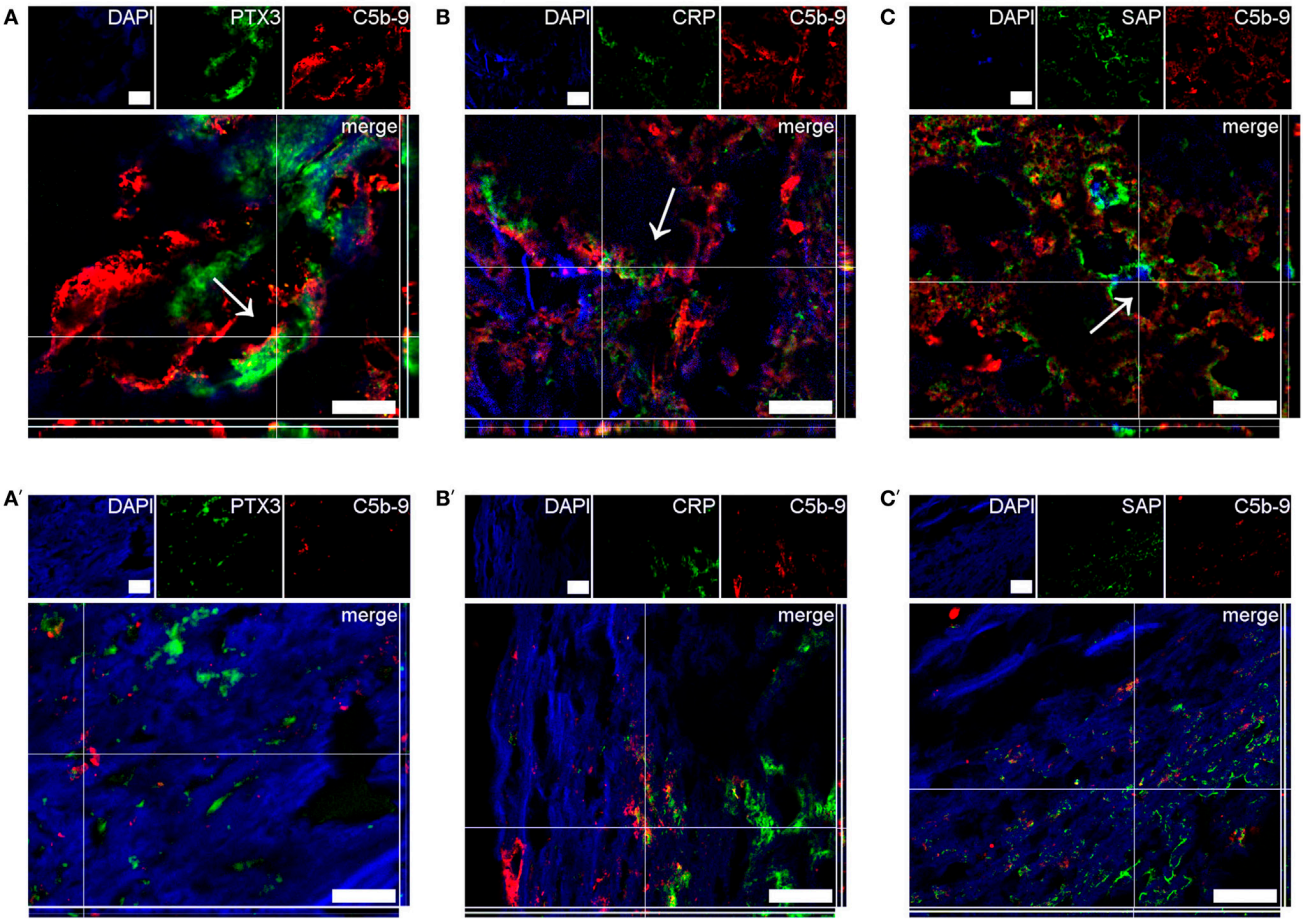
High magnification confocal images showing co-localization of pentraxin 3 (PTX3) **(A,A′)**, C-reactive protein (CRP) **(B,B′)**, and SAP **(C,C′)** with C5b-9 in human atherosclerotic plaques. In the necrotic core **(A–C)**, PTX3, CRP, and SAP (green) surrounded black areas (arrows) likely corresponding to cholesterol clefts, and partially co-localized with C5b-9 (red). In the tunica media **(A′–C′)**, PTX3, CRP, and SAP were present to a lesser extent than in the necrotic core, and showed scarce co-localization with C5b-9. Images in **(A′–C′)** showed the specific signal for each of the proteins obtained after deletion of the collagen-associated unselective fluorescence typical of the tunica media. Images are representative of four plaques analyzed per marker. Scale bars = 20 µm.

To investigate the clinical relevance of the *in vitro* findings, we analyzed the presence of PTX3, CRP, and SAP in human atherosclerotic plaques that were surgically removed from patients with critical stenosis (above 70%) (57). The three proteins were found mainly in the plaque necrotic core (Figure 6), a region rich in cholesterol clefts and fat-laden macrophages (Figure S5 in Supplementary Material), where the complement activation product C5b-9 was also mainly located. We confirmed the protein location after deletion of the collagen-associated unselective signal, which is visible in all three color channels used (exclusion image, Figure 6) and here CRP seemed to be the only pentraxin overlapping with C5b-9. Next, confocal imaging using high magnification was performed to study co-localization of the pentraxins and C5b-9. PTX3, CRP, and SAP were found to surround black non-fluorescent areas in the necrotic core, likely corresponding to cholesterol deposits (Figure S6 in Supplementary Material), where CRP partially co-localized with C5b-9 (Figures 7A–C). PTX3, CRP, and SAP were to a lesser extent present in the tunica media, a region with fewer cholesterol deposits. Even so, CRP appeared to co-localize with C5b-9 (Figures 7A΄–C΄).

## Discussion

Pentraxins are not only a family of acute-phase proteins expressed in response to inflammatory stimuli or tissue damage, but they have also been associated with the development of cardiovascular diseases including atherosclerosis. The pentraxins PTX3, CRP, and SAP interact with PRMs from the complement system, especially C1q. In the present study, we report a novel link between the presence of pentraxins in atherosclerotic lesions and the known complement activation and inflammatory response induced by CC.

The non-glycosylated CRP (61, 62) and the glycoprotein SAP (63, 64) are composed of are composed of protomers organized in a pentameric structure, where five subunits are arranged with radial symmetry (65), whereas the long pentraxin PTX3 has a fourfold symmetry, consisting of two disulfide-linked tertiary rings forming a complex octameric structure (66). PTX3, CRP, and SAP bind to a wide range of ligands including pathogens and altered/damaged self (12). CRP and SAP binding is mostly calcium dependent due to a calcium-binding pocket found in the proteins. PTX3 does not contain this calcium-binding pocket and it has previously been described that purified PTX3 does not bind calcium (20), which might explain some of the different binding specificities of the pentraxins (11). In the binding experiments on CC, binding of purified and native CRP from serum/plasma was calcium dependent, whereas CRP in the systemic inflammation serum pool was not. Also, pSAP bound in a calcium-dependent manner, while SAP in serum/plasma and inflammation serum showed only partially calcium dependency. For PTX3, the opposite was seen—binding of rPTX3 was calcium independent, whereas the native PTX3 was partially calcium dependent. The calcium dependency of native PTX3 could be a result of synergistic interaction with endogenous/native PRMs, e.g., C1q where globular head interactions with ligands are clearly calcium dependent. The possible synergistic interaction with other serum molecules could also explain why a higher amount of rPTX3 compared to native serum PTX3 was required for CC binding. Regarding CRP, our experiments indicate that CRP binding becomes less affected by EDTA when the concentration of CRP increases: NHS vs. inflammation serum and moreover patient 1 (<5 mg/L CRP) vs. 2 and 3 (>100 mg/L CRP) shows that elevated CRP decreases EDTA interference with the binding. It can be speculated whether this is a result of a corresponding increase in calcium-dependent CRP complexes reducing the level of available EDTA to chelate calcium, or whether CRP binding is stabilized by a calcium-independent factor present during inflammation.

Pentraxin 3, CRP, and SAP have previously been shown to interact with the complement PRM C1q. CRP and SAP bind C1q in a highly calcium-dependent manner and interact mainly with the globular head domains of C1q, leading to C1q-mediated complement activation (67, 68). PTX3 binds the globular heads of C1q independent of calcium leading to increased C1q-mediated complement activation (21). Fluid phase interaction of PTX3 with C1q on the other hand inhibits C1q-mediated complement activation, suggesting a dual role for PTX3 in the interaction with the complement system (21). The interaction between rPTX3, pCRP or pSAP, and pC1q used in the binding experiments with CC was characterized in ELISA before use. In agreement with previous literature, our ELISA results showed that C1q binds immobilized PTX3, CRP, and SAP independent of calcium. But, CRP does not bind to immobilized C1q as PTX3 and SAP do (21). Interestingly, our CC binding studies showed that CRP recruits pC1q much more potently to the surface of CC than PTX3 does. Also in UCS, where the level of IgM is much lower than in normal serum, C1q binding was highly increased *via* CRP, but not PTX3. Hence, it appears that CRP can work as an adaptor for C1q in exchange for IgM on the CC. Our previous study supports this finding as we showed that C1q binding to CC is mediated by IgM and not by IgG on the crystal surface (6). The complement activation experiments correspondingly showed that CRP, and not PTX3, increased C5b-9 *via* C1q in UCS. This is central since CRP levels can increase dramatically during inflammation and possibly surpass the IgM-derived complement activation. The concentration of CRP in the complement activation experiment was rather low in order to mimic low grade inflammation. Higher concentrations would possibly have a more potent effect that could mimic acute inflammation *in vivo*. PTX3 increased the binding of C1q in normal serum/plasma and not UCS. Considering the low IgM level in UCS compared to normal serum, this could mean that PTX3 has a positive effect on C1q/IgM complexes. However, it cannot be excluded that an unknown factor found in UCS blocks the interaction between PTX3 and C1q causing the difference between normal serum and UCS.

Preincubation of CC with C1q did not increase the binding of CRP on the CC most likely because the interaction happens *via* the C1q globular head domains. In general, SAP had no effect. Overall these data demonstrate that among the pentraxins, especially CRP may function as an important sensor molecule for the complement system on CC in atherosclerotic plaques. The pentraxins have also been shown to interact with the lectin pathway PRMs ficolin-1, ficolin-2, and MBL (23–26); MBL and ficolin-2 have been shown to activate the complement system on CC and could be a subject for future research (6).

Cholesterol crystals are known to be phagocytosed by granulocytes and monocytes by complement-dependent mechanisms; opsonization of CC by C3b/iC3b leads to phagocytosis *via* CR3 and by binding of PRM to specific receptors (8). Here, we verify previous findings that phagocytosis of CC depends on C1q-mediated complement activation. The pentraxins could have a potential role in directing C1q-mediated complement activation to the CC, in a similar way as natural IgM, leading to removal of CC from atherosclerotic plaques.

In the present study, the interaction between the pentraxins and C1q on the surface of CC was shown by *in vitro* experiments using purified proteins, plasma/serum samples or a whole blood model. To further explore the pathophysiological relevance of these observations, we examined human atherosclerotic plaques for deposition of PTX3, CRP, SAP, and complement activation product C5b-9. PTX3, CRP, and SAP have previously been found in human atherosclerotic lesions (50–53). PTX3 has been shown to be localized in advanced human atherosclerotic lesions, where it was produced by the major cellular components of atherosclerotic plaques macrophages and endothelial cells and to a minor extent by smooth muscle cells (50). CRP has been found in early lesions, co-localized with the complement activation product C5b-9 (51). We found deposition of PTX3, CRP, and SAP in human atherosclerotic plaques, especially in the necrotic core of the lesions, an area rich in cholesterol clefts, but indeed only CRP showed a co-localizing pattern with C5b-9. This strongly supports the idea that CRP-mediated complement activation on CC might be relevant in relation to atherosclerotic processes. Previous studies suggested that CRP-mediated activation of complement is restricted to the early steps of the cascade with little activation of the final product C5b-9 and the inflammatory mediator C5a (69). Bound CRP may also provide a binding site for the soluble control protein factor H, thereby regulating the alternative pathway amplification and C3 convertase. Our results show that CRP co-localizes with C5b-9. However, it is unknown if the C5b-9 in our experiments is indeed activated by CRP or if the factors that under normal circumstances restrict CRP-mediated complement activation to early steps are absent from atherosclerotic plaques.

In conclusion, this study identifies CRP as a strong mediator of complement activation on CC. Since CRP increases vastly during inflammation, complement may exacerbate this condition through CRP and thus play a role in the pathophysiology of atherosclerosis. On the other hand, C1q also mediates phagocytosis of CC and in this way CRP may benefit clearance of CC from the inflamed area. However, in atherosclerosis, immune homeostasis is impaired and likely complement is inappropriately activated, thus contributing negatively to the disease.

## Author Contributions

KP: study design, experimental work, data interpretation, drafting the article, and final approval. SF and AR: experimental work, data interpretation, drafting the article, and final approval. NG, M-OS, M-GS, and PG: study design, data interpretation, critical revision of the article, and final approval. CP and AF: data interpretation, critical revision of the article, and final approval.

## Conflict of Interest Statement

The authors declare that the research was conducted in the absence of any commercial or financial relationships that could be construed as a potential conflict of interest.

## Abbreviations

CCs, cholesterol crystals; CRP, C-reactive protein; CR3, complement receptor 3; C4BP, C4b-binding protein; hsCRP, high-sensitivity CRP; MBL, mannose-binding lectin; NLRP3, nod-like receptor pyrin domain-containing 3; PRM, pattern recognition molecule; SAP, serum amyloid P component; UCS, umbilical cord serum.
