# Supplementary material for: C-Reactive Protein Binds to Cholesterol Crystals and Co-Localizes with the Terminal Complement Complex in Human Atherosclerotic Plaques
Source: Front Immunol. 2017 Aug 29;8:1040. doi: 10.3389/fimmu.2017.01040 (PMC5581807; doi:10.3389/fimmu.2017.01040)
Supplement: Supplementary file 1 [file Supplementary_Material.docx]

Supplementary Material

**C-reactive protein binds to cholesterol crystals and co-localizes with the terminal complement complex in human atherosclerotic plaques**

Katrine Pilely^1^, Stefano Fumagalli^2^, Anne Rosbjerg^1^, Ninette Genster^1^, Mikkel-Ole Skjoedt^1^, Carlo Perego^2^, Angela M. R. Ferrante^3^, Maria-Grazia De Simoni^2^, Peter Garred^1*^

^1^Laboratory of Molecular Medicine, Department of Clinical Immunology, section 7631, Faculty of Health and Medical Sciences, Rigshospitalet, University of Copenhagen, Copenhagen, Denmark, ^2^Istituto di Ricerche Farmacologiche Mario Negri, Department of Neuroscience, IRCCS, Milan, Italy, ^3^Università Cattolica del S.Cuore

Istituto di Patologia Chirurgica Fondazione "Policlinico Universitario A. Gemelli"

Polo Scienze cardiovascolari e toraciche, Roma, Italy

**Corresponding author:**

Peter Garred

e-mail: peter.garred@regionh.dk

**Supplemental materials and methods**

**Western blot of recombinant PTX3**

Recombinant PTX3 (rPTX3) were loaded on a 4–12% (w/v) Bis-Tris polyacrylamide gel under both reducing and non-reducing conditions using the NuPAGE® system (ThermoFisher Scientific). Electrophoresis were run in NuPAGE MOPS running buffer at 150 V for 60 min. Gels was blotted to nitrocellulose membranes (10600096, GE Healthcare) using the Xcell II™ Blot Modul in NuPAGE® transfer buffer. Membranes were washed 2 times in phosphate-buffered saline (PBS) + 0.05% Tween-20 (PBS-T) and blocked for 15 min with PBS-T + 5% skim milk. PTX3 in the membranes was detected using 0.2 µg/mL in house produced biotinylated anti-PTX3 mAb 11-19-66, followed by HRP conjugated streptavidin (RPN1231, GE Healthcare). Membranes were developed with SuperSignal™ West Femto Maximum Sensitivity Substrate (34095, TermoFischer Scientic). Precision Plus Protein All Blue Standards (1610373, Bio-Rad) was used as standard for molecular weight. The membranes were washed in PBS-T between each step.

**PTX3 concentration in plasma and serum samples**

Plasma or serum concentrations of PTX3 were quantified in previously described specific sandwich enzyme-linked immunosorbent assays (ELISAs)(1). In short, ELISA plates were coated with 2 µg/mL in house produced anti-PTX3 mAb 11-19-66 diluted in PBS. Plates were then incubated with plasma or serum samples diluted in PBS-T for 90 min at RT, shaking. Antigen binding was detected using 2 µg/mL biotinylated anti-PTX3 mAb 11-19-20, followed by HRP conjugated streptavidin (RPN1231, GE Healthcare). Plates were developed with TMB one (Kem-En-Tec) and read at 450 nm. Samples were tested in triplicate against a standard pool with known concentration.

**CRP concentration in plasma and serum samples**

CRP plasma or serum concentrations were measured in standard CRP and high-sensitivity CRP (hsCRP) assays.

**Interactions between C1q and the pentraxins PTX3, CRP and SAP in ELISA**

Maxisorp plates (Thermo Scientific, Nunc) were coated with 5 µg/mL purified C1q (pC1q), rPTX3, purified CRP (pCRP), purified SAP (pSAP), or BSA in coating buffer (15 mM Na_2_CO_3_, 35 mM NaHCO_3_,pH 9.6) O/N at 4 °C. Plates were then washed 2 times in PBS-T+ 1% bovine serum albumin (BSA) (PBS-T/BSA) (calcium free conditions) or barbital (5 mM barbital sodium, 145 mM NaCl, 2 mM CaCl_2_, 1mM MgCl_2_, pH 7.4) + 0.05% Tween-20 + 1% BSA (Barbital-T/BSA) (calcium conditions) and blocked for 1h at RT. Plates were then incubated for 1 h at 37 °C with a two-fold dilution of antigen; 20-0 µg/mL rPTX3, pCRP, pSAP or pC1q diluted in PBS-T/BSA or barbital-T/BSA. Plates were then washed and antigen binding was detected using 2 µg/mL specific detection antibodies; biotinylated mouse anti-PTX3 mAb 11-19-20, rabbit anti-CRP pAb (235752, Calbiochem), rabbit anti-SAP pAb (565191, Calbiochem), or rabbit anti-C1q pAb (A0136, Dako) followed by HRP conjugated streptavidin (RPN1231, GE Healthcare) or HRP conjugated swine anti-Rabbit IgG (P0399, DAKO). Plates were developed with TMB one (Kem-En-Tec) and read at 450 nm.

| **Supplemental Table 1. PTX3 and CRP concentrations in plasma and serum samples** | | |
| --- | --- | --- |
| **Sample** | **PTX3 (µg/L)** | **CRP (mg/L)** |
| Hirudin plasma pool (NHP) (n=6 healthy individuals) | < 2 | 0.88 |
| Serum pool (No additive, NHS) (n=6 healthy individuals) | < 2 | 0.93 |
| Patient with systemic inflammation #1 (98089) | 21.7 | 4.0 |
| Patient with systemic inflammation #2 (98067) | 33.0 | 108.7 |
| Patient with systemic inflammation #3 (98109) | 44.3 | 185.8 |
| Serum pool from pt. #1, #2, and #3 (mean, n=3) | 33.0 | 99.5 |


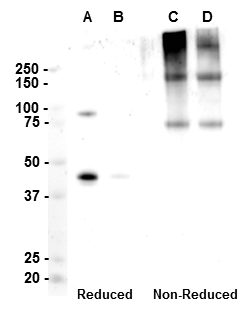


**Supplemental Figure 1. Western blot of purified recombinant PTX3.** Lanes A and B show purified rPTX3 (loading volume 10 ul in A and 5 ul in B) under reducing conditions. The PTX3 monomer has a molecular weight of 45 kDa as previously described by Bottazzi et al.(2). Lanes C and D show purified rPTX3 under non-reducing conditions (loading volume 10 ul in A and 5 ul in B). Latter formation represents the distribution between higher oligomeric forms of PTX3 in the purified recombinant protein.


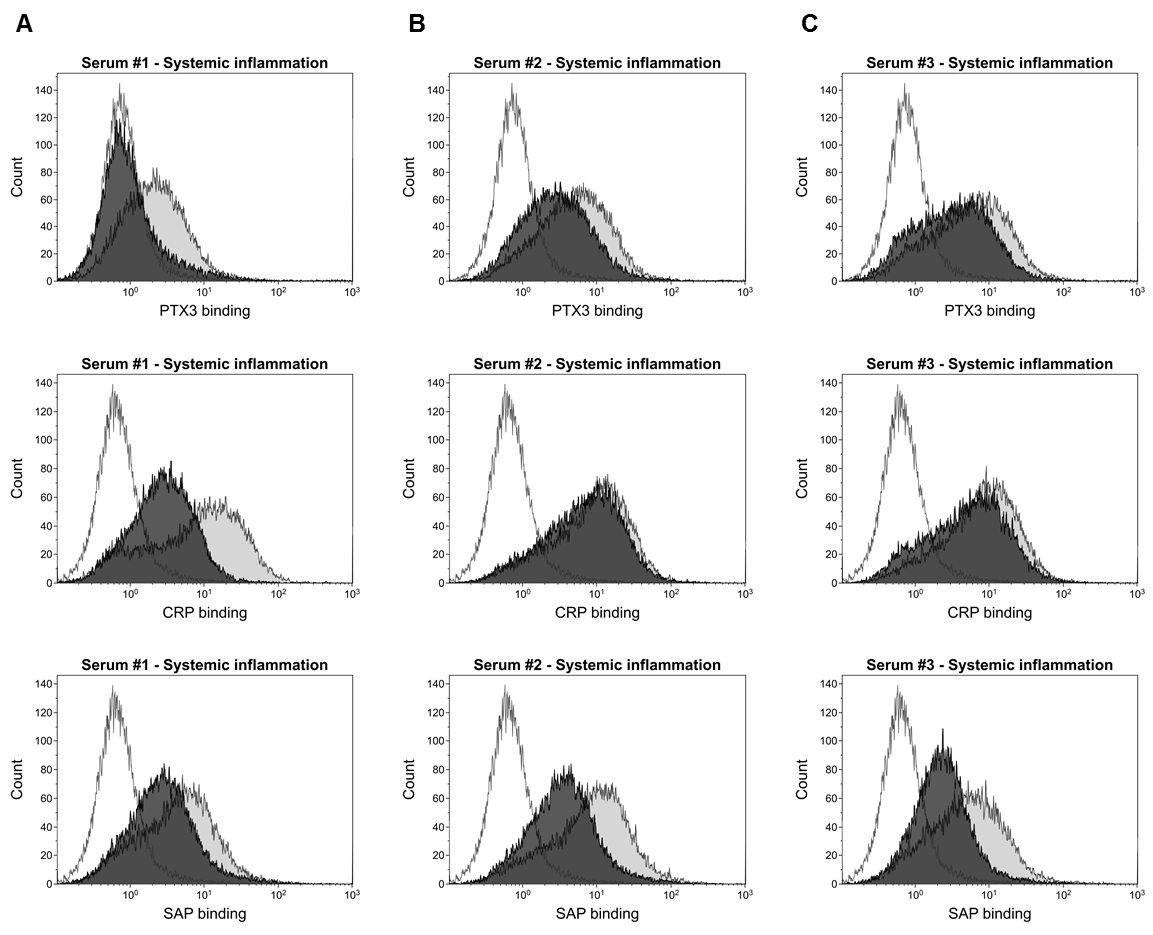


**Supplemental Figure 2. Binding of PTX3, CRP, or SAP to cholesterol crystals from three individuals with systemic inflammation measured by flow cytometry.** **A-C,** Binding of PTX3, CRP and SAP to cholesterol crystals from individual #1 (PTX3 = 21.7 µg/mL, CRP = 4.0 µg/mL) **(A)**, #2 (PTX3 = 33.0 µg/mL, CRP = 108.7 µg/mL) **(B)**, or #3 (PTX3 = 44.3 µg/mL, CRP = 185.8 µg/mL) **(C)** in barbital/BSA (gray) + EDTA (black). Isotype controls are shown as white histograms. Histograms represent one of three independent experiments.


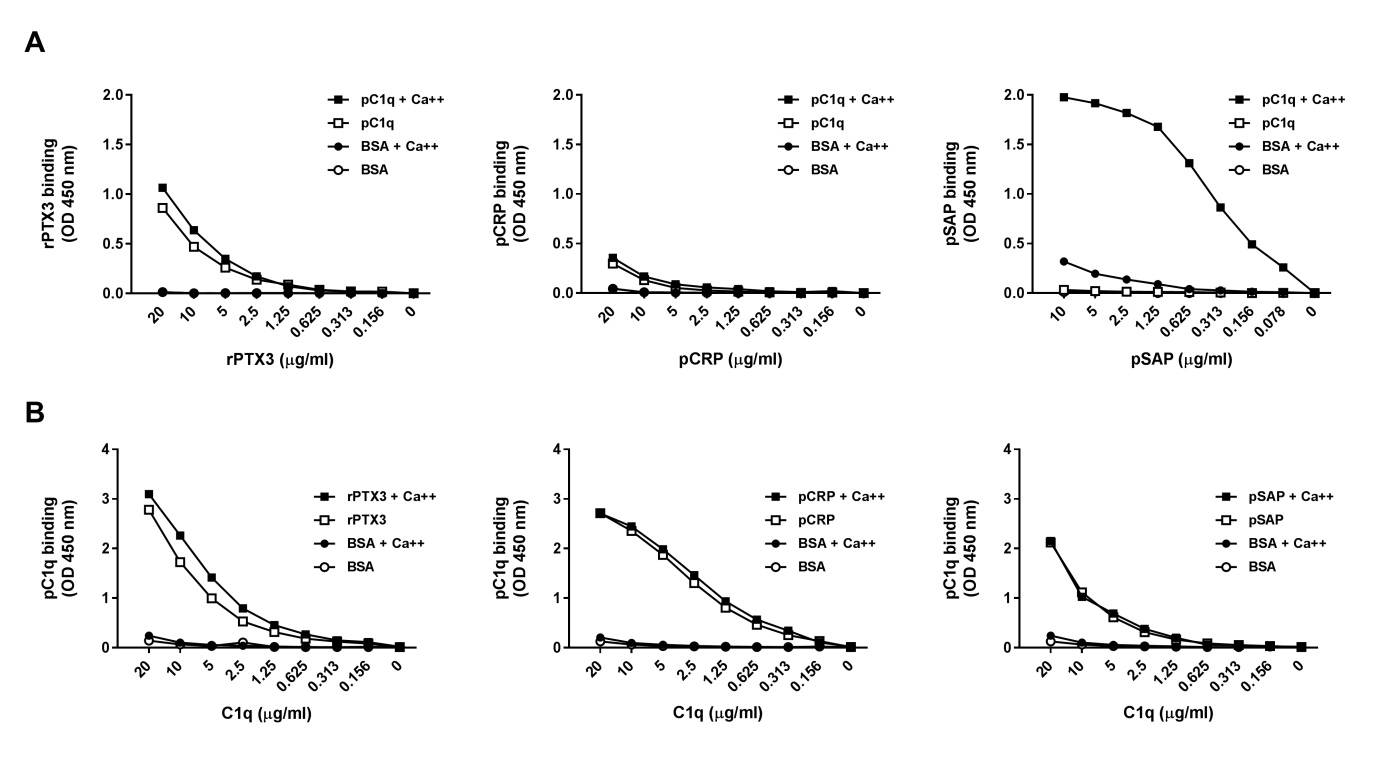


**Supplemental Figure 3. Interaction between C1q and the pentraxins; PTX3, CRP and SAP measured by ELISA.** **A,** Binding of 20-0 µg/mL rPTX3, pCRP or pSAP in the absence or presence of calcium to pC1q or BSA coated on an ELISA plate. **B,** Binding of 20-0 µg/mL pC1q in the absence or presence of calcium to rPTX3, pCRP, pSAP or BSA coated on an ELISA plate. Data represent one of three independent experiments.

**
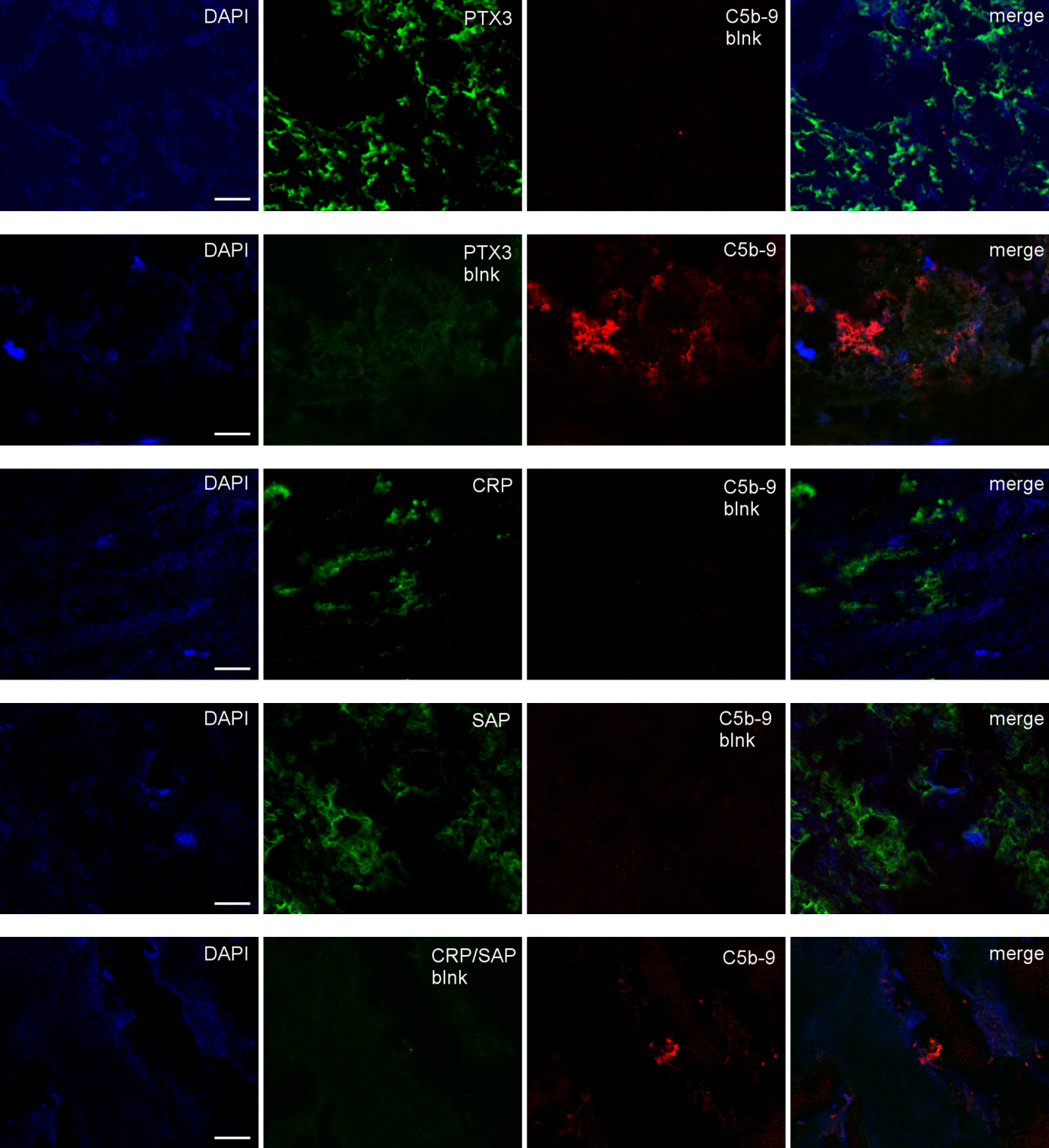
**

**Supplemental Figure 4. Negative controls for the immunofluorescence staining.** Each staining procedure for the double labelling of PTX3, CRP or SAP with C5b-9 did not yield any signal when the primary antibody was omitted (blnk). Scale bars = 20 µm.

**
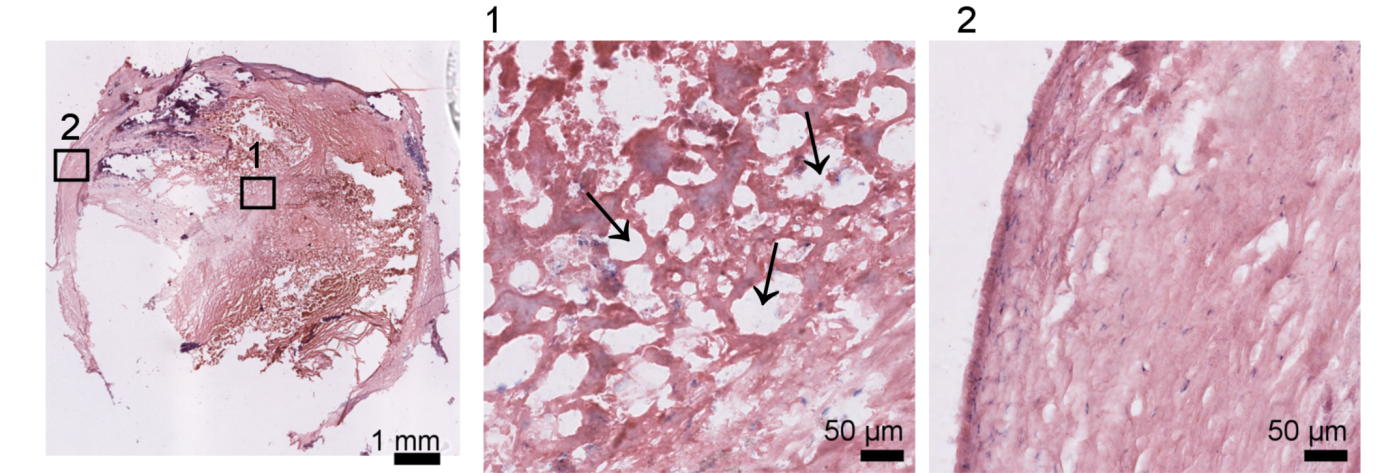
**

**Supplemental Figure 5. Hematoxilin&Eosin staining of the atherosclerotic plaque.** At high magnification the lipid core (insert 1) appears to include the areas corresponding to fat-laden macrophages. These fatty deposits (arrows) likely correspond to the black areas found in the necrotic core, around which PTX3, CRP and SAP deposit. The high magnification of the tunica media is provided in insert 2. Scale bars are indicated on the figure.


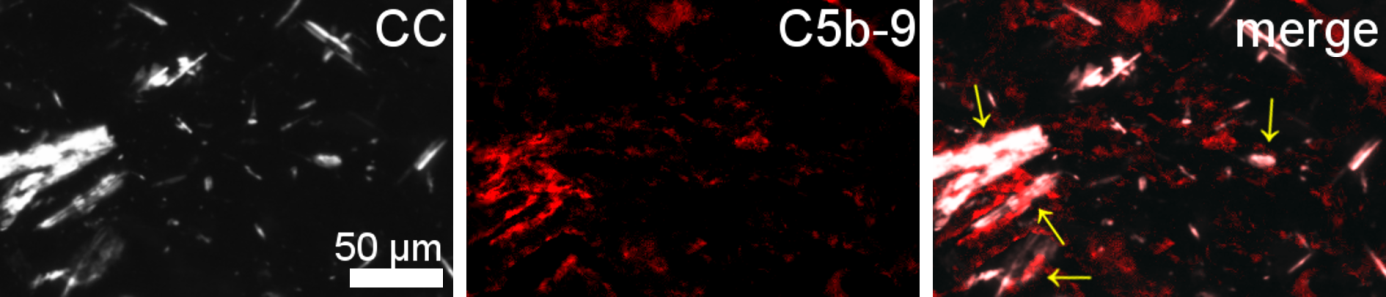


**Supplemental Figure 6. Polarized microscopic image of the necrotic core of a human atherosclerotic plaque.** CCs (white) and C5b-9 (red) in the necrotic core of human atherosclerotic plaques. CCs, imaged using polarized microscopy, are found in the necrotic core of human atherosclerotic plaques. C5b-9 surrounds CCs (yellow arrows in merge) although not exclusively. Scale bar= 50 µm.

**Supplemental references**

1. Bastrup-Birk S, Skjoedt M-O, Munthe-Fog L, Strom JJ, Ma YJ, Garred P. Pentraxin-3 serum levels are associated with disease severity and mortality in patients with systemic inflammatory response syndrome. *PLoS One* (2013) **8**:e73119. doi:10.1371/journal.pone.0073119

2. Bottazzi B, Rie Vouret-Craviari V, Bastone A, De Gioia L, Matteucci C, Peri G, Spreafico F, Pausa M, D ’ettorreʈ C, Gianazza E, et al. Multimer Formation and Ligand Recognition by the Long Pentraxin PTX3 SIMILARITIES AND DIFFERENCES WITH THE SHORT PENTRAXINS C-REACTIVE PROTEIN AND SERUM AMYLOID P COMPONENT. *J Biol Chem* (1997) **272**:32817–32823.
